# Agriculture

**Published:** 1856-06

**Authors:** 


### AGRICULTURE

Pursued with intelligent industry, affords a larger number of high advantages than any other occupation of human life; it strengthens the body, invigorates the mind; and while it refines the sentiments, it purifies the heart, by compelling it to look upward for reliance and help towards Him *who giveth rain and fruitful seasons.* It curbs inordinate ambitions, by yielding a moderate remuneration for toil, while at the same time it imparts a feeling of quiet confidence in the future, from the declaration that while the world stands, *seed time and harvest shall not cease.* The young man brought up to till the soil, begins to feel gradually that the rewards of his toil, are proportioned to his labor, and this imparts by degrees a spirit of self-reliance, which begets independence, and an amount of industrious activities, worth more to that young man, in his after conflicts with the world, than the inheritance of unearned thousands.—Israel Putnam worked faithfully on his father's farm to his full age, and thus secured to himself those self-reliant feelings, which were the germs of all his subsequent distinction.

